# Soil Application of Effective Microorganisms (EM) Maintains Leaf Photosynthetic Efficiency, Increases Seed Yield and Quality Traits of Bean (*Phaseolus vulgaris* L.) Plants Grown on Different Substrates

**DOI:** 10.3390/ijms20092327

**Published:** 2019-05-10

**Authors:** Marcello Iriti, Alessio Scarafoni, Simon Pierce, Giulia Castorina, Sara Vitalini

**Affiliations:** 1 Department of Agricultural and Environmental Sciences, Milan State University, 20133 Milan, Italy; simon.pierce@unimi.it (S.P.); giulia.castorina@unimi.it (G.C.); 2 Department of Food, Environmental and Nutritional Sciences, Milan State University, 20133 Milan, Italy; alessio.scarafoni@unimi.it

**Keywords:** EM technology, food security, sustainable crop production, pulses, biofertilizer, biocontrol agents

## Abstract

EM (effective microorganisms) is a biofertilizer consisting of a mixed culture of potentially beneficial microorganisms. In this study, we investigated the effects of EM treatment on leaf in vivo chlorophyll *a* fluorescence of photosystem II (PSII), yield, and macronutrient content of bean plants grown on different substrates (nutrient rich substrate vs. nutrient poor sandy soil) in controlled environmental conditions (pot experiment in greenhouse). EM-treated plants maintained optimum leaf photosynthetic efficiency two weeks longer than the control plants, and increased yield independent of substrate. The levels of seed nutritionally-relevant molecules (proteins, lipids, and starch) were only slightly modified, apart from the protein content, which increased in plants grown in sandy soil. Although EM can be considered a promising and environmentally friendly technology for sustainable agriculture, more studies are needed to elucidate the mechanism(s) of action of EM, as well as its efficacy under open field conditions.

## 1. Introduction

The global food security challenge is straightforward: by 2050, the world must feed around 9 billion people, and, consequently, the demand for food will increase by 60%. Therefore, progress towards food security requires that food is available, accessible, and of sufficient quantity and quality to ensure good nutritional outcomes, particularly in protracted socioeconomic crises [1]. In this alarming scenario, new approaches for crop production are more than ever of paramount importance.

Biostimulants, including plant-growth promoting microorganisms, have been shown to increase plant nutrient uptake, growth, and yield via different underlying mechanisms such as changes in soil structure, nutrient solubility, root growth and morphology, plant physiology, and symbiotic relationships. In addition, they can improve the plant tolerance to abiotic stresses, as well as the resistance to pathogens [2,3]. EM (effective microorganisms) is an environmentally friendly technology consisting of a fermented mixed culture of coexisting and mutually compatible microorganisms in an acidic medium. This biofertilizer contains up to 80 different species belonging to five main groups of microorganisms, including photosynthetic bacteria (*Rhodopseudomonas palustris*, *Rhodobacter sphaeroides*), lactic acid bacteria (*Lactobacillus plantarum*, *L. casei*, *Streptococcus lactis*), yeasts (*Saccharomyces cerevisiae*, *Candida utilis*), actinomycetes (*Streptomyces albus*, *S. griseus*), and fermenting fungi (*Aspergillus oryzae*, *Penicillium* spp., *Mucor hiemalis*) [4,5]. Similarly to other biostimulants, EM can positively affect plant nutrition, modify root morphology, and selectively change the rhizosphere–microbial community structure. The use of EM as an amendment has been reported for different crops by some authors to enhance soil fertility, increase crop yield, and control plant diseases [5,6,7,8,9,10,11,12]. However, in cotton plants, application of EM alone did not increase yield significantly over control, though in combination with organic matter it resulted in a 44% increase in yield over control [13]. Similarly, in green manure amendment, EM application resulted in a significant decline of 23% in grain yield of mung bean, while it significantly increased grain yield by 24% and 46% in farmyard manure and NPK fertilizer amendments, respectively [7]. In an open field experiment over two years, EM alone or in combination decreased tomato yield by 27–49% [14]. 

The effects of EM on photosynthesis have also been investigated. EM treatment increased the photosynthesis rate in cabbage plants, as well as stomatal conductance and intracellular CO_2_ concentration [15]. Similarly, photosynthetic efficiency increased in periwinkle plants after EM application [11]. Nonetheless, little is known about the possible variations of the nutritional value of grains from plants treated with this liquid microbial inoculant, nor the physiological mechanisms underlying any variation. We hypothesize that increased yield and nutritional quality of bean (*Phaseolus vulgaris* L.) occur and are associated with maintenance of photosynthetic efficiency. Additionally, we hypothesize that yield increases are evident when EM is applied even on nutrient poor substrates. Thus, we investigated the effects of EM on leaf in vivo chlorophyll *a* fluorescence of photosystem II (PSII), seed yield, and macronutrient content of bean plants grown in two different substrates.

## 2. Results

**Figure 1:**
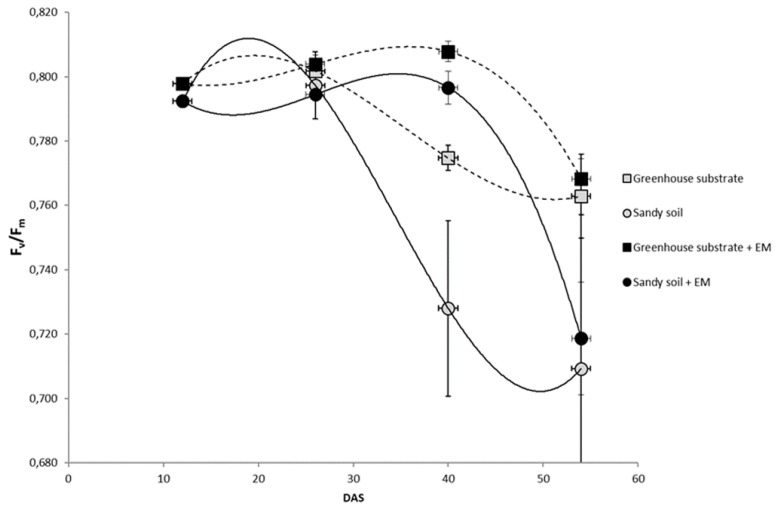
Effects of EM (effective microorganisms) treatments on maximum efficiency of photosystem II (F_v_/F_m_) of bean plants grown on greenhouse substrate and sandy soil. DAS, days after sowing. Results are expressed as mean (*n* = 15) and error bars indicate the standard deviation (SD).

The ratio of variable to maximal fluorescence (F_v_/F_m_), which reflects the maximal photochemical yield of PSII centers, is highly correlated with the quantum yield of net photosynthesis of treated and untreated leaves. Forty days after sowing, differences were significant among treatments and substrates. In particular, F_v_/F_m_ remained at optimal levels (~0.83) for at least two weeks longer for EM-treated plants, regardless of soil (Figure 1). By the time of plant senescence (at 53 DAS, days after sowing), differences in photochemical yield of PSII were determined mainly by substrate, being particularly low on sandy soil compared to the richer substrate.

**Figure 2:**
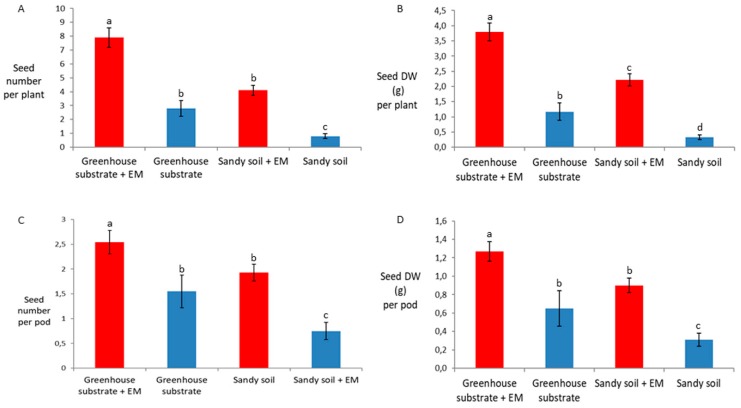
Seed yield. Effects of EM treatments on (**A**) seed number per plant, (**B**) seed dry weight (DW) per plant, (**C**) seed number per pod, and (**D**) seed DW per pod. Bean plants were grown on greenhouse substrate and sandy soil. Results are expressed as mean (*n* = 15), and error bars indicate the SD. Significant differences were accepted at *p* < 0.05 and represented by different letters, according to Tukey’s HSD test.

**Figure 3:**
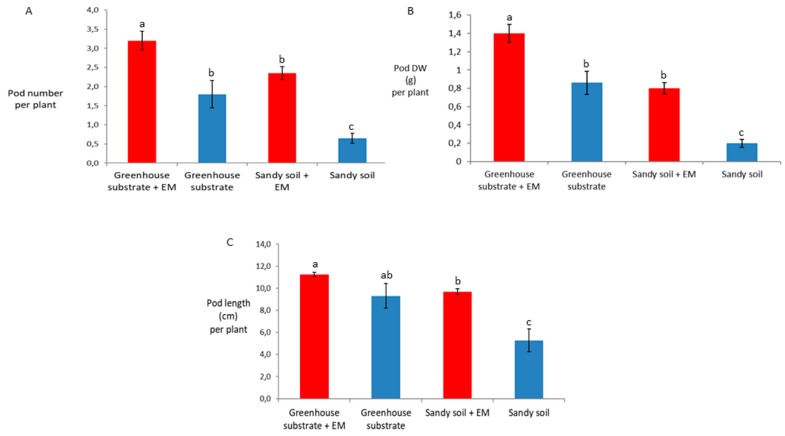
Pod yield. Effects of EM treatments on (**A**) pod number per plant, (**B**) pod dry weight (DW) per plant, and (**C**) pod length. Bean plants were grown on greenhouse substrate and sandy soil. Results are expressed as mean (*n* = 15), and error bars indicate the SD. Significant differences were accepted at *p* < 0.05 and represented by different letters, according to Tukey’s HSD test.

EM application significantly increased all the seed yield properties, i.e., seed number per plant, seed dry weigh (DW) per plant, seed number per pod, and seed DW per pod independently of substrate (Figure 2A–D). Similar results were obtained for the pod yield parameters, apart from the pod length, which was not significantly different between EM-treated and control plants grown in greenhouse substrate (Figure 3A–C).

As far as the seed quality was concerned, the results of the present study (Table 1) indicated that the substrate markedly affected bean composition. In particular, plants grown on sandy soil accumulated less proteins and starch, whereas lipid content was not affected. EM treatment only slightly modified the concentrations of all seed macronutrients, apart from the protein content, which was strongly increased in plants grown in sandy soil.

**Figure 4:**
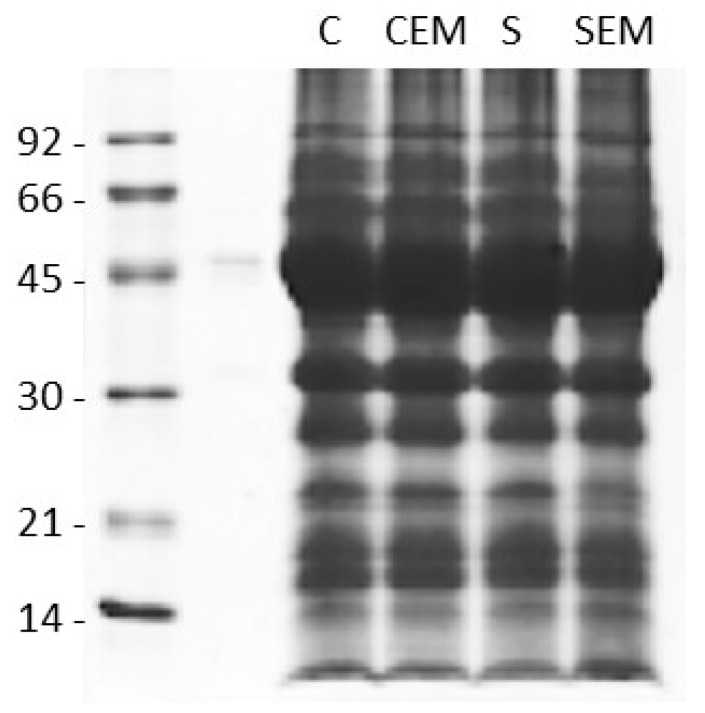
SDS-PAGE (sodium dodecyl sulphate-polyacrylamide gel electrophoresis) of total protein extracts of seeds from bean plants grown on standard or sandy soils, as such or following EM treatment. See the text for experimental details. C: greenhouse substrate; CEM: greenhouse substrate + EMs; S: sandy soil; SEM: sandy soil + EMs.

From a qualitative point of view, no difference of the protein profiles between the treatments was evidenced by electrophoretic analysis (Figure 4).

The amount of resistant starch, which is the starch fraction that is not hydrolysable by human enzymes in the small intestine, depends on the type of soil and was not influenced by treatment with EM. The non-resistant starch portion was not modified. Interestingly, the water content of the grains from plants treated with EM was lower than that of the control plants (about 5% and 7%, respectively) (Table 1). 

Table 2 illustrates the metal ion content in the seeds of bean plants. The results for iron and phosphorus are particularly relevant, both positively influenced by treatment with EM. Indeed, iron contents were highest for bean plants grown in sandy soils with EM treatment (41.6 ± 0.91 mg kg^-1^), which was almost twice the iron content of beans grown on greenhouse substrate without EM (Table 2). Phosphorus contents increased by a quarter with EM treatment (i.e., an increase of 25.9% and 24.0% on greenhouse substrate and sandy soil, respectively; Table 2). Zinc contents exhibited a significant increase with EM treatment on sandy soil, but EM treatment was associated with significantly lower zinc contents on greenhouse substrate (Table 2).

## 3. Discussion

In our experimental conditions, EM application was found to significantly increase yield in bean plants even when grown on poor, sandy soil, thus showing promising potential as a biostimulant. EM treatment was also found to modify seed macronutrient contents, notably increasing protein contents on sandy soil, decreasing lipid contents, and generally increasing iron and phosphorus contents, although many other nutrient contents did not change. Changes in yield and nutrient contents were associated with a maintenance of photochemical efficiency, indicating a lack of constraints to primary metabolism (i.e., less stress).

The first finding, that yield increased with EM treatment, is in agreement with previous studies on bean plants grown under saline conditions. Indeed, EM treatment is known to alleviate salinity stress by modifying a number of physiological processes involved in stress tolerance. Nutrient uptake, relative water content, membrane integrity, levels of soluble sugars, free amino acids, osmoprotectants, reactive oxygen species (ROS), and scavenging capacity increased, whereas lipid peroxidation, ROS production, and electrolyte leakage decreased in salt-stressed plants treated with EM [10,16,17]. In addition, EM treatment under salinity stress increased shoot height, shoot dry weight per plant, leaf area, leaf number per plants, root length, root dry weight per plant, seed number per plant, and seed weight per plant, as well as protein content in seeds and N, P, and K concentrations in seeds and shoots [10,17]. In bean plants, EM was also applied as a biocontrol agent against *Rhizoctonia solani*, significantly decreasing both the severity and incidence of infection. Furthermore, EM treatment significantly increased total plant dry weight and leaf area [6].

The finding that EM treatment modified seed total protein content for plants grown in sandy soil has previously been reported [17]. However, EM treatment decreased the lipid content, regardless the substrate. Overall, our results suggest that EM treatment increases the protein content to the detriment of lipids in poorer soil. In general, EM treatment favored a higher accumulation of metal ions relevant for human nutrition. Iron, calcium, sodium, copper, and phosphorus were the most influenced, as previously reported [10].

Nevertheless, available nutrients in the soil can drive different behaviors in particular microbial consortia. Particularly, varying concentrations of soil N would result in either more amino acids or proteins, depending on the soil microbial communities. In these terms, the differences observed in seed yield and protein content might be due to the diverse N content in the substrates. Indeed, sandy soil was devoid of N. Very recently, the plant growth promoting effects of selected microbial (fungal and bacterial) single-strain inoculants vs. microbial consortium products were compared under real production conditions in large-scale tomato cultivation systems, exposed to different environmental challenges. In a greenhouse (a stress-protected production system), microbial single-strain inoculants and consortium products exhibited very similar stimulatory effects on plant growth and yield. Conversely, higher performance of the microbial consortium products was recorded under more extreme environmental conditions in an open-field drip-fertigated tomato production system in the Negev desert (Israel), where plants were exposed to multiple stresses (high temperatures and radiation intensities, sandy soil, high soil pH, and low soil fertility and organic matter content, as well as limited water availability and P supply) [3]. In addition, we have to take into account that substrate pH represents another relevant issue. Soil pH determines both the soil microbial communities and the interactions that they can establish with the rhizospheres of plants, as well as the solubility of metals. Indeed, soil acidity favors iron and zinc solubility, and, consequently, the plant uptake of these metals. Therefore, even if the pH values of greenhouse substrate and sandy soil were 6.0 and 7.5, respectively, the increased iron and zinc levels measured in seeds cannot be solely attributed to the EM. Noteworthy, the biofertilizer is applied in acidic solution (pH 3.4), optimal for EM activation (the activation process is finished at pH range of 3.2–3.5).

Finally, we can speculate on the possible mechanisms involved in the observed effects, focusing on two phenomena relevant for plant growth and productivity: the maintenance of PSII efficiency and P uptake. Maintenance of photochemical efficiency for several extra weeks (with respect to controls) indicates that EM-treated plants experienced less stress, with fewer impacts on primary metabolism, and thus were effectively able to grow for longer. As treatments represented modification of edaphic conditions (soil type and soil microbial community) it is reasonable to speculate a mechanism whereby EM addition improved the availability of mineral resources for plant growth. Whatever the precise mechanism(s), further studies are needed in order to ascertain the real efficacy of EM under open field conditions, where plants are exposed to multiple co-occurring biotic and abiotic stresses, by assessing the root length and morphology, the levels of ROS and scavengers, stress-related secondary metabolites, and phytohormones.

## 4. Materials and Methods

### 4.1. Plant Material and EM Treatments

Bean (*Phaseolus vulgaris* L. cv. Borlotto Nano Lingua di Fuoco) seeds were surface-sterilized with 1% sodium hypochlorite solution for 1 min, rinsed in sterile distilled water, air-dried, and then planted in 22 cm (5 L) pots (1 seed per pot). Two different substrates were used: (i) a greenhouse substrate (Vigorplant^®^ SER CA-V7) (*n* = 30) and (ii) a sandy soil (Termit^®^, 0.6 mm Ø) (*n* = 30) (Table 3). Plants were grown in a greenhouse at 24 ± 2 °C temperature, 60 ± 5% relative humidity (HR) and a 16 h/8 h light/dark period. The photon flux density at plant height was 500 µmol Q m^−2^ s^−1^ =, provided by incandescent lamps. Approximately 10–12 days after seeding, when the primary leaves were completely expanded, plants (*n* = 15 per substrate) were treated every 7 days by soil drench with EM-1^®^ (kindly provided by Punto EM s.r.l., http://www.italiaem.it/Home/em.html), activated according to the supplier information (pH 3.4), until the end of flowering (BBCH 79). The experiment ran for 80 days.

### 4.2. Leaf Chlorophyll a Fluorescence Analysis

In vivo chlorophyll *a* fluorescence of photosystem II (PSII) was excited and detected with a pulse amplitude modulation fluorimeter (OS1-FL, Opti-sciences, Hudson (NH), WI, USA), on the adaxial surface of interveinal regions of the two youngest fully expanded leaves, before treatment (T_0_), i.e., 12 days after seeding (DAS), and then every 14 days until the end of flowering (BBCH 79). Before the beginning of each measurement, leaves were dark-adapted for 40 min [18]. The maximum quantum yield (Φ_PSII_) was assessed as (F_m_ − F_0_)/F_m_ = F_v_/F_m_. F_0_, defined as dark-adapted minimal fluorescence, is the initial level of the chlorophyll *a* fluorescence when all PSII reaction centers are open, and was measured under low ambient background light. F_m_ is the dark-adapted maximal fluorescence when all PSII reaction centers are closed, and was measured by applying a saturating actinic light pulse (15,000 µmol Q m^−2^ s^−1^ for 2 s duration). Finally, F_v_ is the result of F_m_ − F_0_, and represents the variable fluorescence in dark-adapted leaves: a value of ~0.83 represents maximal efficiency, whereas lower values indicate stress [19].

### 4.3. Effects of EM on Plant Production

At harvest maturity, fruits were collected and oven dried for 48–72 h until constant weight. The following yield ratios and productivity parameters were measured: (i) number and dry weight of seeds per fruit; (ii) number and dry weight of fruits per plant; (iii) number and dry weight of seeds per plant; and (iv) length of fruits.

### 4.4. Seed Composition Analyses

Water content was determined as follows. About 1 gram of bean flour, placed in a glass capsule, was exactly weighed and then heated to 110 °C until a constant weight was reached. Before each measurement, capsules were closed with a lid and left to cool to room temperature. Samples were analyzed in triplicate. Resistant and non-resistant starch amounts were determined according with AOAC Method 2002.02 AACC Method 32-40.01 with a Megazyme (Wicklow, Ireland) assay kit (K-RSTAR), following the manufacturer’s instructions. Beans were ground finer than 60 mesh, and flour samples of about 1 g were analyzed in triplicate. For protein analysis, beans were ground finer than 60 mesh. One gram of flour was extracted (1:20 *p*/*v*) with a solution containing 7 M Urea, 2 M Thiourea and 2% CHAPS for 3 hours at RT. Samples were then centrifuged at 10,000× *g* for 20 min. The supernatant was collected and kept at −20 °C for further analyses. The quantification of the protein content in solution was determined according to Bradford [20]. BSA (bovine serum albumin) was used as the standard. SDS-PAGE (sodium dodecyl sulphate-polyacrylamide gel electrophoresis) was carried out as described by Laemmli [21] on 12.5% polyacrylamide gels, using a mini-Protean III electrophoretic cell (Bio-Rad, Segrate, Milan, Italy). Runs were carried out at a constant 16 mA/gel. Polypeptides were visualized by Coomassie Brilliant Blue staining (Bio-Rad). Lipids were determined gravimetrically. One gram of bean flour was exactly weighed and extracted with hexane (150 mL) using a Soxhlet device for 8–10 hours. The flour was then placed in an electric oven at 50 °C to allow the evaporation of the solvent till a constant weight was reached. Samples were analyzed in triplicate.

### 4.5. Metal Ion Contents

Samples (0.25 g) were ground with dry ice and digested using a microwave digestor system (Anton Paar Multiwave-Eco, Rivoli, Torino, Italy), in Teflon tubes with 10 mL of 65% HNO_3_. A two-step power ramp was applied (step 1: 200 W in 10 min, maintained for 5 min; step 2: 650 W in 10 min, maintained for 15 min). Samples were diluted 1:40 with Milli-Q water, and ion concentrations were measured by inductively coupled plasma (ICP) mass spectroscopy (Bruker AURORA M90 ICP-MS, Milan, Italy).

### 4.6. Statistical Analysis

All experiments were carried out in triplicate in a completely randomized design, and results are reported as mean ± standard deviation (SD) or mean ± standard error of the mean (SEM). Variable distribution was assessed by the Kolmogorov–Smirnov normality test. Data of each parameter were subjected to one-way analysis of variance (ANOVA), to compare the effects of treatments, and comparison among means was determined according to Tukey’s honestly significant difference (HSD) test. Significant differences were accepted at *p* < 0.05 and represented by different letters.

## Figures and Tables

**Table 1:** Composition of seeds from bean plants grown in different soils, not treated or treated with EM. Data are expressed as g of nutrients per 100 g of dry seed matter. The water content of the sample is reported in the last row. In each row, values (mean ± SEM) with the same letters are not significantly different between treatments of the same substrate (*p* ≤ 0.05).

Constituents	Greenhouse Substrate		Sandy Soil	
	CTRL	EM	CTRL	EM
**Proteins**	25.02 ± 3.61 a	25.02 ± 0.53 a	23.47 ± 1.51 a	26.53 ± 1.14 b
**Lipids**	8.99 ± 0.02 a	7.57 ± 0.03 b	9.0 ± 0.03 a	7.04 ± 0.02 c
**Total starch**	37.01 ± 1.70 a	38.47 ± 1.85 a	33.00 ± 1.33 b	32.29 ± 1.42 b
**Resistant**	32.56 ± 1.49 a	33.58 ± 1.61 a	28.66 ± 1.15 b	28.27 ± 1.24 b
**Non-resistant**	4.45 ± 0.21 a	4.89 ± 0.24 a	4.37 ± 0.18 a	4.02 ± 0.18 a
**Water**	10.17 ± 0.01 a	9.45 ± 0.02 b	10.19 ± 0.03 c	9.68 ± 0.02 d

**Table 2:** Metal ion contents of seeds from bean plants grown in different soils, not treated or treated with EM. Data are expressed as mg of metal per kg of bean seed. In each row, values (mean ± SEM) designated with the same letters are not significantly different between treatments of the same substrate (*p* ≤ 0.05).

Metal Ions	Greenhouse Substrate		Sandy Soil	
	CTRL	EM	CTRL	EM
**Calcium**	250.23 ± 7.81 a	230.45 ± 6.93 b	525.63 ± 19.95 c	564.05 ± 21.65 c
**Copper**	1.69 ± 0.09 a	1.49 ± 0.11 a	3.50 ± 0.25 b	2.97 ± 0.18 b
**Iron**	22.43 ± 0.56 a	24.33 ± 0.53 a	32.20 ± 0.84 b	41.57 ± 0.91 c
**Magnesium**	507.72 ± 9.63 a	594.40 ± 9.85 b	611.65 ± 9.76 b	597.26 ± 9.11 b
**Manganese**	4.28 ± 0.07 a	4.67 ± 0.11 b	5.08 ± 0.12 c	5.32 ± 0.09 c
**Phosphorus**	1469.44 ± 25.91 a	1984.14 ± 22.67 b	1433.33 ± 21.48 a	1888.12 ± 19.88 b
**Potassium**	4487.46 ± 55.64 a	5875.30 ± 58.35 b	5718.39 ± 60.12 b	5329.87 ± 56.93 c
**Sodium**	6.35 ± 0.12 a	14.28 ± 0.13 b	10.66 ± 0.12 c	16.01 ± 0.12 d
**Zinc**	13.69 ± 0.66 a	8.96 ± 0.35 b	11.61 ± 0.43 c	17.59 ± 1.21 d

**Table 3:** Chemico-physical characteristics of the substrates.

**Substrate**	**Properties**					
	**Components**	**pH**	**Total N** **(%, Dry Weight)**	**Electrical Conductivity** **(dS m^−1^)**	**Apparent Density** **(kg m^−3^)**	**Total Porosity** **(%*v*/*v*)**
**Greenhouse substrate (potting soil unfertilized)**	Peat	6.0	1.4	0.25	120	90
**Substrate**	**Properties**					
	**Components**	**pH**		**Ø (mm)**	**SiO_2_ (%)**	**Mohs scale**
**Sandy soil (unfertilized)**	Sand	7.5	0	0.6	75–80	6
